# Quantitative Susceptibility Mapping Reveals an Association between Brain Iron Load and Depression Severity

**DOI:** 10.3389/fnhum.2017.00442

**Published:** 2017-08-29

**Authors:** Shun Yao, Yi Zhong, Yuhao Xu, Jiasheng Qin, Ningning Zhang, Xiaolan Zhu, Yuefeng Li

**Affiliations:** ^1^Department of Radiology, The Affiliated Hospital of Jiangsu University Zhenjiang, China; ^2^Department of Research and Development, Magnetic Resonance Innovations Inc. Detroit, MI, United States; ^3^Department of Neurology, The Affiliated Hospital of Jiangsu University Zhenjiang, China; ^4^Department of Gynaecology and Obstetrics, The Fourth Affiliated Hospital of Jiangsu University Zhenjiang, China

**Keywords:** depression, quantitative susceptibility mapping, iron, putamen, thalamus

## Abstract

Previous studies have detected abnormal serum ferritin levels in patients with depression; however, the results have been inconsistent. This study used quantitative susceptibility mapping (QSM) for the first time to examine brain iron concentration in depressed patients and evaluated whether it is related to severity. We included three groups of age- and gender-matched participants: 30 patients with mild-moderate depression (MD), 14 patients with major depression disorder (MDD) and 20 control subjects. All participants underwent MR scans with a 3D gradient-echo sequence reconstructing for QSM and performed the 17-item Hamilton Depression Rating Scale (HDRS) test. In MDD, the susceptibility value in the bilateral putamen was significantly increased compared with MD or control subjects. In addition, a significant difference was also observed in the left thalamus in MDD patients compared with controls. However, the susceptibility values did not differ between MD patients and controls. The susceptibility values positively correlated with the severity of depression as indicated by the HDRS scores. Our results provide evidence that brain iron deposition may be associated with depression and may even be a biomarker for investigating the pathophysiological mechanism of depression.

## Introduction

Iron, as an electron facilitator, serves many brain functions including myelin production, neurotransmitter synthesis, oxygen transport and electron transfer (Moos and Morgan, 2004; Stankiewicz et al., 2007; Hare et al., 2013). Both postmortem and noninvasive iron-sensitive MRI methods have previously shown that the concentration of iron in the brain is not uniform (Hallgren and Sourander, 1958; Li et al., 2014; Ramos et al., 2014). Moreover, within the normal ageing brain, iron accumulates in the deep gray brain matter structures such as the putamen (PU), globus pallidus (GP), thalamus (THA) and caudate nuclei (CN; Hallgren and Sourander, 1958). Regional excessive iron contributes to the continual generation of radical species and toxic free radicals (Emerit et al., 2001), which are harmful to the motor and cognitive functions of the brain, as they damage dopamine synthesis (Yehuda and Youdim, 1989). Such activity leading to neuronal cell death is thought to be a result of the ageing process as well as a variety of neuropsychiatric diseases, such as Alzheimer’s disease, Parkinson’s disease and multiple sclerosis (Núñez et al., 2012; Moon et al., 2016; Zhang et al., 2016). In addition to the mechanisms thought to be associated with age- and disease-related iron accumulation, recent work on neurodegeneration with brain iron accumulation (NBIA) indicated that iron misregulation, such as increased iron accumulation, might be modulated by genetic factors (Gregory and Hayflick, 2005; Heidari et al., 2016).

Depression is a neuropsychosis that is a highly prevalent and disabling mental condition and is a leading cause of disease burden worldwide. There have been various explanations of the pathophysiology of depression; however, a consensus has yet to be reached. Recently, there has been increasing interest in a possible protective and modifiable role of metallic elements, especially Zn and Fe, due to their antioxidant activity (Szkup et al., 2017). In depression, peripheral blood markers such as serum ferritin concentrations are most often applied to assess iron levels because they are relatively simple and convenient (Zhu et al., 2016). However, given the many factors interfering with iron transport, uptake and storage, serum ferritin concentrations are easily confounded, thereby not accurately reflecting total body iron content (Nielsen et al., 2000).

The best way to accurately and directly detect iron is through histological measurement. Iron can also be estimated *in vivo* by noninvasive MRI, which is based on proton relaxation rates or the magnetic field changed by the paramagnetic properties of iron. A variety of MRI methods using gradient echo sequences were proposed to represent iron concentration in the brain (Aquino et al., 2009). However, phase signal is easily affected by the formation of the magnetic field distribution because of the nonlocal nature of the magnetic field distribution (Schweser et al., 2011). Quantitative susceptibility mapping (QSM) has overcome these limitations because it is independent of field strength and object shape (Wharton and Bowtell, 2010). In addition, compared with phase imaging or other MR methods, QSM typically provides a higher sensitivity and specificity for detecting brain iron (Liu et al., 2015; Wang and Liu, 2015). With such impressive performance, QSM has been applied to detect brain iron concentration in Huntington’s disease, Parkinson’s disease and multiple sclerosis (Zhang et al., 2016; Eskreis-Winkler et al., 2017).

Here, we use the QSM method to investigate the brain iron concentration in patients with depression. We hypothesized that in patients with depression, increased magnetic susceptibility of the deep gray matter nuclei is correlated with depression severity. Moreover, we also examined variables of Hamilton Depression Rating Scale (HDRS) scores among patients with different degrees of depression to identify the latent relation between susceptibility and HDRS score.

## Materials and Methods

### Participants

According to the Chinese version of the Modified Structured Clinical Interview for DSM-IV, 47 outpatients were recruited from the Department of Psychiatry. Inclusion criteria included no history of neurological illnesses, severe diseases or head injury. All individuals underwent MRI at 3T and received extended diagnostic examinations such as clinical history, vascular factors tests (including blood pressure), and a 17-item HDRS. Other necessary information was acquired. Three patients were excluded due to their MR images being unavailable. Thus, 44 individuals were included in this study. According to the HDRS score, the patients were separated into two groups: mild-moderate depression (MD, 7 < HDRS < 19, *n* = 30) and Major Depression Disorder (MDD, HDRS ≥ 19, *n* = 14).

Additionally, 20 control subjects were recruited, who were strictly screened by the inclusion criteria and ability to undergo MRI.

This study was carried out in accordance with the recommendations of the Ethical Committee of the Affiliated Hospital of Jiangsu University with written informed consent from all subjects. All subjects gave written informed consent in accordance with the Declaration of Helsinki. The protocol was approved by the Ethical Committee of the Affiliated Hospital of Jiangsu University.

### MR Image Acquisition

All scanning images were performed with a 3T scanner (Siemens Magnetom Trio) using an 8-channel head coil. A 3D gradient-echo sequence was applied to perform QSM. The imaging parameters were: repeat time (TR) = 46 ms with echo time (TE) = 18 ms, slice thickness = 2 mm, flip angle = 7°, bandwidth/pixel = 120 Hz/pixel, FOV = 25 cm, matrix size = 448 × 448 and scan time = 6 min.

### Image Processing

**Figure 1:**
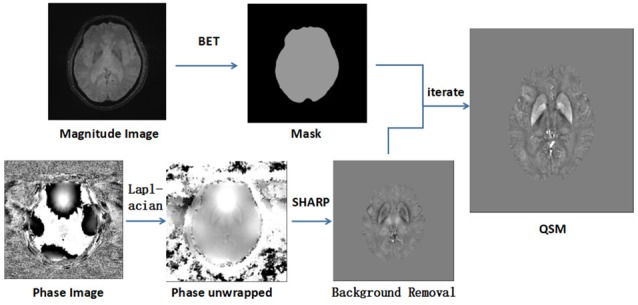
This image shows the image processing steps for quantitative susceptibility mapping (QSM). Phase unwrapped using the Laplacian algorithm and removed unreliable voxels with a mask obtained from magnitude images by SHARP. With an iterate method, quantitative susceptibility map images were reconstructed.

Brain masks were calculated using the magnitude of the images with the Bet algorithm in FSL with the threshold set to 0.2 (Smith, 2002). Phase images were unwrapped with the Laplacian approach (Schofield and Zhu, 2003). After unwrapping, the background field was removed with the SHARP filtering method using a kernel size with a maximum radius of 6 mm and a singular value decomposition threshold of 0.05 (Schweser et al., 2011). Finally, we used an iterative algorithm to generate QSM images (Tang et al., 2013; Figure 1). The original susceptibility maps were derived from the following equation (for a left-handed system; Haacke et al., 2010):

$$\text{Δχ}=FT^{-1}\;[FT(-P_{cor}/(\gamma B_{0}TE))/\left(\frac{1}{3}-\frac{k_{z}^{2}}{k_{x}^{2}+k_{y}^{2}+k_{z}^{2}}\right)]$$

where *P*_cor_ is the phase distribution of unwrapped and background-field corrected phase map; *γ* is the gyromagnetic ratio for hydrogen protons; *B*_0_ is the main magnetic field strength; *TE* is the echo time; and *k*_x_, *k*_y_, *k*_z_ are coordinates in *k*-space.

### Data Analyses

**Figure 2:**
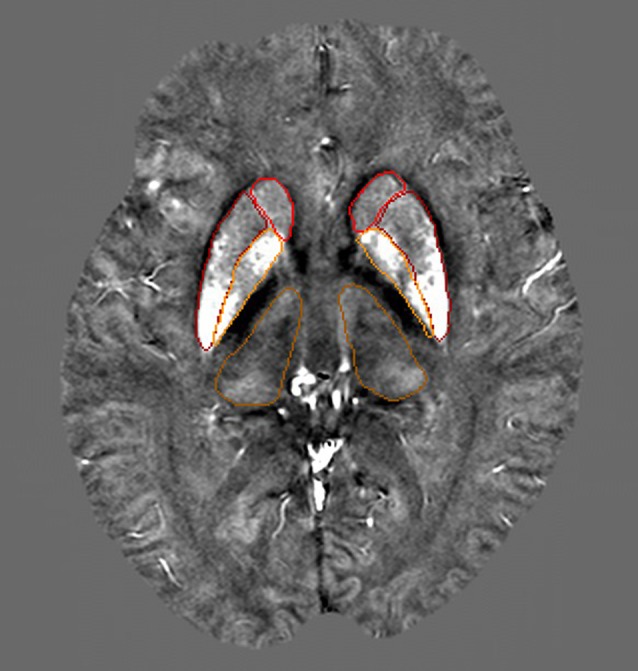
This image shows four selected regions of one QSM image slice from a 40-year-old subject. Red: caudate nucleus (CN; head), Dark red: putamen (PU), Orange: globus pallidus (GP), Dark orange: thalamus (THA).

The GM nuclei in this study were segmented manually on the QSM images based on the gray matter nuclei anatomical features for each case, including GP, putamen (PUT), caudate nucleus (CN) and thalamus (THA). Figure 2 shows one axial slice of a QSM image with the nuclei segmented from one individual. Multiple slices of a region of interest (ROI) were drawn to access the average susceptibility value by two neuroradiologists who were blinded to the diagnoses and clinical information. These neuroradiologists have more than 5 years of experience in image processing. In our study, we used SPIN software (Magnetic Resonance Innovations Inc., Detroit, MI, USA) to directly estimate the local susceptibility values of ROIs without reference regions for each subject.

### Statistical Analysis

Statistical analysis was performed using SPSS (v.22.0; SPSS Inc., Chicago, IL, USA). The Kolmogorov-Smirnov test was used to analyze the normality of the data. ANOVA was applied to compare the patient demographics, vascular factors, HDRS among MD, MDD and control subjects. The difference of QSM values among three groups was compared by using ANCOVA, adjusted for age and sex, and *post hoc* Bonferroni was used for correction of multiple comparisons. All results of multiple comparisons presented in this study had been corrected. The relationship between iron accumulation and HDRS was analyzed by the Pearson correlations. A value of *p* < 0.05 was considered statistically significant.

## Results

### Data Analysis of Demographics

Demographic characteristics of the study participants are summarized in Table 1. Given iron deposition increases with normal ageing, the age-matched controls and depression groups revealed no differences in age. The HDRS scores were higher in the MDD group and significantly differed from those in the MD group (in MD: 12.73 ± 3.06, in MDD: 24.35 ± 3.49, *p* < 0.001). There were no differences among these groups in years of education, gender or vascular factors.

**Table 1:** Demographic and clinical characteristics of all groups in this study.

	Control (*n* = 20)	MD (*n* = 30)	MDD (*n* = 14)	*p*
Age, years	41.95 ± 10.35	43.36 ± 12.28	45.14 ± 8.83	0.709
Education, years	10.15 ± 2.66	9.10 ± 2.53	8.57 ± 2.79	0.198
Gender (female, %)	12 (60)	14 (46.6)	6 (42.8)	0.544
Hypertension (%)	2 (10%)	5 (17%)	3 (21%)	0.064
HDRS		12.73 ± 3.06	24.35 ± 3.49	<0.001

*Data represent the means ± standard deviation. MD, mild-moderate depression; MDD, major depression disorder*.

### Susceptibility Values of QSM Imaging between Groups

**Figure 3:**
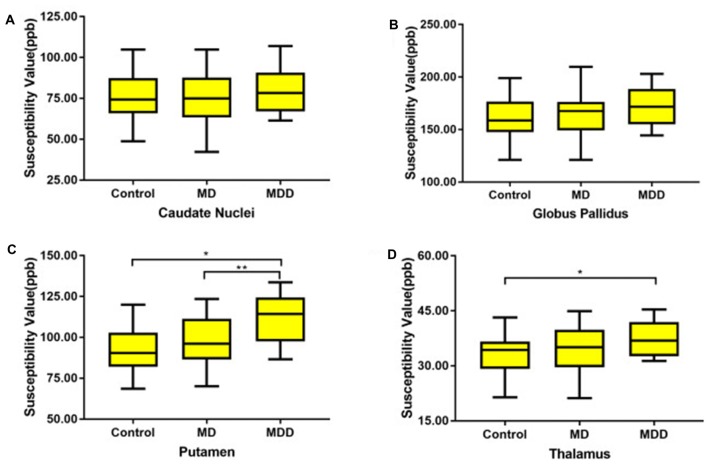
The average susceptibility of iron concentrations for bilateral gray matter region (**A**: CN, **B**: GP, **C**: PU **D**: THA). MD: mild-moderate depression, MDD: major depression disorder. Note: *Post hoc* comparisons: * means MDD vs. Control (in putamen, *p* < 0.001; in thalamus, *p* < 0.05, ** means MDD vs. MD (in putamen, *p* < 0.05).

**Figure 4:**
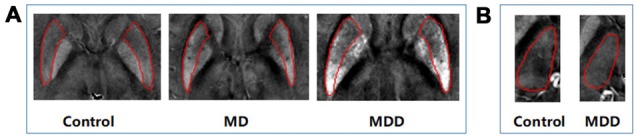
Differences in QSM images of bilateral PUT **(A)** and left thalamus **(B)**. MD: mild-moderate depression, MDD: major depression disorder.

The magnetic susceptibility value of all subjects in each group were quantitatively measured and summarized in Table 2. Differences in magnetic susceptibility values among the control, MD and MDD subjects were assessed using ANCOVA after controlling for gender and age. Among these three groups, the average susceptibility values of the bilateral hemisphere in MDD showed the most in all ROIs (Figure 3). In Figure 3, in these selected regions, the GP had a higher QSM value than other nuclei. There was a significant increase in the average susceptibility values was found in the PUT and THA (MDD vs. Control in putamen, *p* < 0.001, and in thalamus, *p* < 0.05; MDD vs. MD in putamen, *p* < 0.05; Figure 3). Compared to control, MDD had a significant increase in the bilateral putamen (left, *p* < 0.001; right, *p* < 0.001) and the left thalamus (*p* = 0.023; Figure 4). Between MD and MDD, the values differed significantly in the bilateral putamen (left, *p* = 0.022 and right, *p* = 0.015) while did not differ in thalamus (left, *p* = 0.083, right, *p* = 0.384).

**Table 2:** Data represent means ± standard deviation.

QSM (ppb)	Control (*n* = 20)	MD (*n* = 30)	MDD (*n* = 14)	Significant differences (*P* value)
CN	75.98 ± 13.56	75.33 ± 15.05	79.93 ± 14.58	Not significant (0.671)
Left	75.74 ± 13.54	74.33 ± 17.44	80.28 ± 14.24	Not significant (0.548)
Right	76.23 ± 13.63	77.01 ± 15.30	79.58 ± 14.98	Not significant (0.729)
GUP	161.03 ± 20.68	164.69 ± 18.81	172.80 ± 17.24	Not significant (0.232)
Left	161.24 ± 20.67	163.41 ± 18.41	173.30 ± 17.47	Not significant (0.141)
Right	160.83 ± 20.73	165.96 ± 20.72	172.31 ± 17.59	Not significant (0.222)
PUT	91.20 ± 13.58	97.63 ± 15.61	111.20 ± 14.58	MDD vs. Control (<0.001)
				MDD vs. MD (0.019)
Left	91.18 ± 13.78	98.04 ± 15.59	110.95 ± 15.21	MDD vs. Control (<0.001)
				MDD vs. MD (0.022)
Right	91.22 ± 13.51	97.23 ± 16.25	111.45 ± 14.35	MDD vs. Control (<0.001)
				MDD vs. MD (0.015)
THA	33.14 ± 5.51	34.44 ± 6.09	37.21 ± 4.74	MDD vs. Control (0.038)
Left	33.87 ± 5.96	35.76 ± 5.70	38.41 ± 5.03	MDD vs. Control (0.023)
Right	32.40 ± 5.19	32.13 ± 6.55	36.02 ± 4.09	Not significant (0.191)

*MD, mild-moderate depression; MDD, major depression disorder*.

### Correlation between Susceptibility Values and HDRS

**Figure 5:**
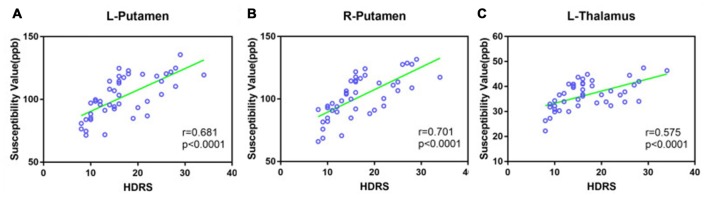
Scatter plot illustrating the relationship between HDRS and susceptibility values in MD and MDD (**A**: Left putamen, **B**: right putamen, **C**: left thalamus). MD: mild-moderate depression, MDD: major depression disorder.

A Pearson correlation was used to investigate the relationship between QSM values in the bilateral putamen and the left thalamus, and HDRS scores were obtained for both MD and MDD (Figure 5). HDRS was significantly correlated with susceptibility values in the bilateral PUT (left, *r* = 0.681, *p* < 0.0001; right, *r* = 0.701, *p* < 0.0001) and left THA (*r* = 0.575, *p* < 0.001).

### Validation of QSM Data

To validate whether magnetic susceptibility data provide a quantitative measure of iron, linear regression was applied to find the correlation between the susceptibility of deep brain gray matter nuclei from the control group in the present study and the iron distribution in postmortem samples as reported by Hallgren and Sourander (1958). A correlation (*r* = 0.97) was found after exclusion of the caudate nucleus because Hallgren and Sourander (1958) did not provide the scatterplots of caudate iron distribution in their article.

## Discussion

To the best of our knowledge, this is the first assessment of brain iron distribution among depressed patients. In the present study, we used QSM to analyze the differences of brain iron concentration between patients with depression (MD and MDD) and healthy controls of the same age range. Our data showed that brain iron abnormally accumulated in iron abundant regions, namely, the CN, putamen, GP and thalamus, as detected by QSM in depressed patients. The susceptibility values in MDD patients were higher than that in control or MD subjects. However, susceptibility values did not differ between MD patients and controls. The highest concentration of iron deposition was in GP while the region with the lowest concentration of iron was in the THA. Additionally, iron accumulation was related to HDRS, which may implicate iron metabolism in the pathophysiology of depression.

Previous studies commonly used serum or plasma ferritin to elucidate the link between depression and body iron, but regretfully, the results are still inconclusive. Some researchers found that higher body iron was associated with more depressive symptoms and serum ferritin levels were significantly higher at admission in post-stroke depression patients (Richardson et al., 2015; Zhu et al., 2016). Some studies indicated that lower levels of serum ferritin concentrations had a higher prevalence of depressive symptoms and these depressive symptoms concomitantly occurred among patients with anemia (Yi et al., 2011). Interestingly, it was also reported that depressive symptoms were not correlated with ferritin levels and that there was no relationship between maternal iron status and postpartum depression (PPD; Stewart and Hirani, 2012; Su et al., 2016). Another study about PPD found that early iron supplementation in mothers with PPD significantly improves the iron stores and even causes a significant improvement in PPD (Sheikh et al., 2017). These contradictory findings could be explained by the vulnerability of serum ferritin regarding iron deficiency and normal iron stores, especially in the liver (Nielsen et al., 2000). Thus, there is a need for a more accurate method to represent body iron in depressed patients. Recently, a novel noninvasive MR measure called QSM was proposed to overcome this limitation by detecting iron content in the brain.

It is well known that the local magnetic field homogeneity can be changed due to the paramagnetic properties of tissue iron. The MR method is used as a noninvasive measure to assess brain iron content *in vivo*. Previous studies using phase imaging or susceptibility weighted imaging had found that abnormal iron deposition in the brain occurred in some neuropsychiatric diseases (Haacke et al., 2009; Walsh et al., 2013). Although phase images have high spatial resolution and require less time for scanning, the inferiority of phase imaging is obvious. The low spatial frequency signals of the phase image are lost through high-pass filtering, leaving mostly rapid spatial-varying components. Furthermore, the appearance of phase images is highly vulnerable to nonlocal field influence, which is probably caused by vessels and calcifications (Marques et al., 2009). QSM successfully avoids these limitations because of its unique algorithms. Compared with phase imaging, the QSM method provides more accurate measurements of the deposited iron (Reichenbach, 2012). Therefore, QSM can be used to not only assess important tissue functions and disease but also improve patient care (Wang et al., 2017).

In our study, QSM values of all analyzed regions increased, particularly in the bilateral PUT and left THA in MDD patients, when compared with controls. Although the mechanism of iron accumulation in depression remains unclear, iron overload might contribute to depressive diseases. On one hand, the abnormal deposition of iron might be the result of the depression. Many fMRI studies have suggested that the basal nuclei displayed structural transformation as well as dysfunction in depression (Sacchet et al., 2017; Zhao et al., 2017). More and more evidence suggests that these changes to the basal nuclei, which are associated with motor function, and associative and limbic circuits, were related to the destruction of the dopaminergic system (Schroll and Hamker, 2016; Markett et al., 2017). However, the structural changes may be far behind the functional loss of dopaminergic neurons (Volkow et al., 1996). Dopamine plays a key role in the pathogenesis of depression (Grace, 2016). Brain iron is essential in the synthesis and metabolism of dopamine and thus, there are high levels of iron in the basal nuclei (Zucca et al., 2017). On the other hand, excess iron can be harmful, generating toxic reactive oxygen species and facilitating a neurotoxic process, leading to highly reactive dopaminergic neurons, toxic quinones and even death via the Fenton reaction (Paris et al., 2005). Elevated iron levels also promote the aggregation of some proteins linked to neurodegenerative disorders such as α-synuclei (Li et al., 2010).

We also found GP has the highest susceptibility value among all groups. This is consistent with previous studies using QSM, which explored iron concentrations in PD and MS (Langkammer et al., 2013; He et al., 2015). Several results suggest that individuals with high iron concentration in the GP have a higher risk of iron-overload induced motor function decline (Li et al., 2015). However, the magnetic susceptibility in GP was not significantly correlated with HDRS here. Rather, a significant association with HDRS remained for the PUT and THA. We found the susceptibility values of the bilateral putamen in MDD or MD was positively related with HDRS. Previous studies have shown that the putamen, making up the striatum, is related to motor and cognitive functions (Herrero et al., 2002). The dysfunction of the putamen is known to be associated with loss of dopaminergic neurons within the cortical-striatum-thalamocortical circuit (Moos and Morgan, 2004). The output of the putamen can be abnormally modulated by dopaminergic inputs. Some MRI findings also claimed that the volume of the putamen was altered in depression (Lu et al., 2016), which is in line with our results. With respect to the thalamus, it is thought to be implicated in the pathophysiology of MDD and is currently drawing sustained attention (Kong et al., 2013; Brown et al., 2017). Recent research has documented that abnormal thalamocortical connectivity, specifically abnormal thalamo-temporal and thalamo-somatosensory connectivity, was found in MDD (Brown et al., 2017). A post-mortem study has demonstrated that patients with major depression had more neurons in the thalamic nuclei compared with control samples (Young et al., 2004). Our data showed that the susceptibility significantly increased in the left thalamus, which corroborated previous MR results that described the presence of structural abnormalities in the left thalamus among patients with depression (Lu et al., 2016; Li et al., 2017).

This study has several limitations. One limitation is that only four selected gray matter regions were analyzed. Although iron is abundant in these regions, and the structures displayed well in QSM images, there are other regions that could be assessed. Another limitation is the lack of pathologically confirmed patients. However, all of the patients were strictly screened with both comprehensive neuropsychological assessments and MRI. Moreover, our study is limited by the relatively small number of patients with depression and controls. Therefore, a longitudinal study with a large sample size and more analyzed regions is needed to further elucidate the mechanism of iron in depression.

## Conclusion

We used QSM to explore the pattern of brain iron accumulation in depression for the first time. We found increased iron accumulation mainly in the putamen and thalamus nucleus in depressed patients and related this to the severity of depression based on HDRS. Our results indicate the role of excess brain iron in deep gray matter in depression. This suggests iron may be a potential biomarker for further understanding the pathophysiological mechanism of depression.

## Author Contributions

SY, YL and XZ designed the study and conducted the data analysis. SY wrote the article. YX, JQ and NZ organized the study. YX, JQ, NZ and YZ supported the data analysis. YZ gave technical support. All authors were critically involved in the theoretical discussion and performing of the experiments. All authors read and approved the final version of the manuscript.

## Conflict of Interest Statement

The authors declare that the research was conducted in the absence of any commercial or financial relationships that could be construed as a potential conflict of interest.
